# Cervicitis as a Clinical Indicator of Gonococcal and Chlamydial Infections in Pregnancy

**DOI:** 10.1155/S1064744995000585

**Published:** 1995

**Authors:** Susan L. Jackson, Nan G. O'Connell, Joseph F. Borzelleca, Mara J. Dinsmoor, David E. Soper

**Affiliations:** 1 Department of Obstetrics and Gynecology Medical College of Virginia Virginia Commonwealth University Richmond VA USA; 2 Department of Obstetrics and Gynecology Desk A81 The Cleveland Clinic Foundation 9500 Euclid Avenue Cleveland OH 44195 USA

## Abstract

*Objective:* We undertook the present study to attempt to apply clinical indicators predictive of cervical infection in nongravid populations with either *Neisseria gonorrhoeae* or *Chlamydia trachomatis* to our pregnant population and to determine the significance of the clinical diagnosis of “cervicitis.”

*Methods:* A retrospective chart review of all pregnant women with a final diagnosis of cervicitis who were seen in the Medical College of Virginia obstetrical emergency room was performed during the period of September 1991 to December 1992.

*Results:* Given the diagnosis of cervicitis in our emergency department, we found that the clinical examination predicted cervical infection with *N. gonorrhoeae* or *C. trachomatis* in only 20% of the pregnant women. Gravidas with chlamydial infections were younger (20.1 ± 3.7 years) compared with gravidas not infected (23.2 ± 5.4 years) (P < 0.0001). They were also more likely to have a diagnosis of lower urinary-tract infection [relative risk (RR) 2.89, 95% confidence interval (CI) 1.42–5.85].

*Conclusions:* The clinical indicators of cervical infection with *C. trachomatis* and *N. gonorrhoeae* were unreliable.


Infectious Diseases in Obstetrics and Gynecology 3:184–188 (1995)
© 1996 Wiley-Liss, Inc.

### Cervicitis as a Clinical Indicator of Gonococcal and Chlamydial Infections in Pregnancy

Susan L. Jackson, Nan G. O'Connell, Joseph F. Borzelleca, Mara J. Dinsmoor, and David E. Soper

*Department of Obstetrics and Gynecology, Medical College of Virginia, Virginia Commonwealth University, Richmond, VA*

### Abstract

*Objective:* We undertook the present study to attempt to apply clinical indicators predictive of cervical infection in nongravid populations with either *Neisseria gonorrhoeae* or *Chlamydia trachomatis* to our pregnant population and to determine the significance of the clinical diagnosis of "cervicitis."

*Methods:* A retrospective chart review of all pregnant women with a final diagnosis of cervicitis who were seen in the Medical College of Virginia obstetrical emergency room was performed during the period of September 1991 to December 1992.

*Results:* Given the diagnosis of cervicitis in our emergency department, we found that the clinical examination predicted cervical infection with *N. gonorrhoeae* or *C. trachomatis* in only 20% of the pregnant women. Gravidas with chlamydial infections were younger (20.1 ± 3.7 years) compared with gravidas not infected (23.2 ± 5.4 years) ($P < 0.0001$). They were also more likely to have a diagnosis of lower urinary-tract infection [relative risk (RR) 2.89, 95% confidence interval (CI) 1.42–5.85].

*Conclusions:* The clinical indicators of cervical infection with *C. trachomatis* and *N. gonorrhoeae* were unreliable. © 1996 Wiley-Liss, Inc.

Key words

Sexually transmitted diseases, endocervicitis, *Neisseria gonorrhoeae, Chlamydia trachomatis*

Screening populations of nongravid women reveals that several variables are predictive of *Chlamydia trachomatis* infections: age younger than 24 years, intercourse with a new partner in the preceding 2 months, purulent or mucopurulent cervical exudate, endocervical bleeding induced by swabbing, and lack of contraception or use of a nonbarrier method.[1] Several studies in developing countries aimed at identifying those variables that would support the clinical diagnosis of chlamydial or gonococcal infections in pregnant women have produced mixed results.[2,3]

To date, there have been no published studies in industrialized countries to examine the value of clinical indicators in predicting cervical infections in pregnant women. We undertook the present study to attempt to apply clinical indicators predictive of cervical infections with either *Neisseria gonorrhoeae* or *C. trachomatis* in nongravid populations to our pregnant population and to determine the significance of the clinical diagnosis of "cervicitis."

### SUBJECTS AND METHODS

A retrospective chart review was carried out of all pregnant women seen in the Medical College of Virginia obstetrical emergency room with a final diagnosis of cervicitis during the period of September 1991 to December 1992. This time was chosen as a convenience sample to include approximately

Address correspondence/reprint requests to Dr. Susan L. Jackson, Department of Obstetrics and Gynecology, Desk A81, The Cleveland Clinic Foundation, 9500 Euclid Avenue, Cleveland, OH 44195.

Received October 25, 1995

Accepted January 11, 1996

250 women. Cervicitis was defined as the presence of endocervical mucopus, leukorrhea (>10 WBCs/hpf), or cervical friability (cervical bleeding induced with a swab). Lower urinary-tract infection, as diagnosed by urinalysis, was defined as the presence of leukocytes, bacteria, nitrites, or erythrocytes in the absence of large numbers of epithelial cells on a clean-catch or catheterized urine specimen. All women were interviewed and examined by either an obstetrical resident or an obstetrics and gynecology nurse practitioner. The data collected on all patients included age, race, estimated gestational age, gravidity, parity, presenting complaints, and history of sexually transmitted diseases (STDs) including chlamydial and gonococcal infections. In addition, information was obtained regarding the presence of endocervical mucopus or cervical friability, direct smear of endocervical secretions for WBCs, results of wet mount examination of pooled vaginal fluid for the presence of WBCs and *Trichomonas vaginalis,* and any other diagnoses assigned at the time of discharge.

Each patient underwent a speculum examination with collection of endocervical specimens for *N. gonorrhoeae* culture and for chlamydial antigen detection (Chlamydiazyme, Abbott Laboratories, Chicago, IL). Each endocervical culture for *N. gonorrhoeae* was inoculated onto Martin Lewis Transgrow medium (Becton Dickinson, Cockeysville, MD) and then incubated at 35°C in a 5% carbon dioxide atmosphere. A positive culture was presumptively identified by a Gram's stain of the colonies and a positive oxidase test. The endocervical specimens for *C. trachomatis* were collected using the STD-EZE collection kit (Abbott Laboratories). The presence of the chlamydia antigen was determined in the clinical microbiology laboratory using the Chlamydiazyme enzyme-linked immunosorbent assay (ELISA) test following the manufacturer's directions.

A statistical analysis was performed utilizing the Student's t-test for continuous variables. The categorical variables were analyzed utilizing the chi-square or Fisher exact test when appropriate. $P < 0.05$ (using a two-tailed statistical test) was considered significant.

### RESULTS

Two hundred fifty-five pregnant women with a diagnosis of cervicitis were seen between September 1991 and December 1992. The results of gonorrhea and chlamydia testing were available for 251. Three of the samples obtained for chlamydia testing read as "inconclusive" were not included, leaving 248 patients for analysis. Ten (4%) women tested positive for *N. gonorrhoeae* and 42 (17%) tested positive for *C. trachomatis.* Of those infected with gonorrhea, 3 (30%) were coinfected with chlamydia. Because the clinical diagnosis of cervicitis was so poorly predictive of the presence of either *N. gonorrhoeae* or *C. trachomatis,* we then examined the individual clinical findings to determine whether any one was a better predictor of the presence of infection in pregnant women. Of the patients with gonococcal infections, 40% had cervical friability, 50% had endocervical mucopus, and 80% had leukorrhea. In those with chlamydial infections, friability was noted in 55%, endocervical mucopus in 55%, and leukorrhea in 88%. Friability was present in 127 patients, of whom 4 (3%) tested positive for *N. gonorrhoeae* and 23 (18%) tested positive for *C. trachomatis.* Mucopus was present in 119 patients, of whom 5 (4%) tested positive for *N. gonorrhoeae* and 23 (19%) tested positive for *C. trachomatis.* Leukorrhea was seen in 211 patients, of whom 8 (4%) tested positive for *N. gonorrhoeae* and 37 (17%) tested positive for *C. trachomatis.*

The mean age of patients having a positive Chlamydiazyme was significantly younger compared with those having a negative test: $20.1 \pm 3.7$ vs. $23.2 \pm 5.4$ years ($P < 0.0001$) (Table 1). Thirty-six women (86%) with *C. trachomatis* were younger than 24 years. There were no significant differences between groups in gestational age, gravidity, parity, history of STDs, complaints of dysuria, abdominopelvic pain, vaginal bleeding, or vaginal discharge (Tables 1, 2). There were also no statistical differences in the incidence of cervical friability, endocervical mucopus, or WBCs or *T. vaginalis* on wet mount (Table 3). There was an association between the presence of a positive Chlamydiazyme test and a final discharge diagnosis of lower urinary-tract infection [$P = 0.006$; relative risk (RR) 2.89, 95% confidence interval (CI) 1.42–5.85].

### DISCUSSION

STDs are common complications of pregnancy, particularly in indigent urban populations, with *N. gonorrhoeae* and *C. trachomatis* being among the most common organisms encountered.[4] *C. trachomatis* is

TABLE 1. Comparison of characteristics of pregnant women with cervicitis, with and without gonoccocal or chlamydial infection[a]

| | *N. gonorrhoeae* | | | *C. trachomatis* | | |
|---|---|---|---|---|---|---|
| Characteristic | Positive (N = 10) | Negative (N = 238) | *P* | Positive (N = 42) | Negative (N = 206) | *P* |
| Age (years) | 22.2 ± 5.0 | 22.7 ± 5.2 | NS | 20.1 ± 3.7 | 23.2 ± 5.4 | <0.0001 |
| EGA (weeks) | 18.7 ± 11.3 | 17.9 ± 9.7 | NS | 17.6 ± 9.8 | 18.0 ± 9.8 | NS |
| Gravidity | 3.3 ± 1.8 | 3.2 ± 2.0 | NS | 3.0 ± 2.3 | 3.2 ± 1.9 | NS |
| Parity | 1.6 ± 1.4 | 1.4 ± 1.5 | NS | 1.3 ± 1.4 | 1.5 ± 1.5 | NS |
| Abortus | 0.7 ± 1.1 | 0.8 ± 1.1 | NS | 0.7 ± 1.4 | 0.8 ± 1.1 | NS |

[a]EGA = estimated gestational age; NS = not significant.

TABLE 2. Comparison of symptoms of pregnant women with cervicitis, with and without gonoccocal or chlamydial infection[a]

| | *N. gonorrhoeae* | | | *C. trachomatis* | | |
|---|---|---|---|---|---|---|
| Symptom | Positive (N = 10) | Negative (N = 238) | *P* | Positive (N = 42) | Negative (N = 206) | *P* |
| Bleeding | 2 (20%) | 112 (47%) | NS | 17 (40%) | 97 (47%) | NS |
| Discharge | 4 (40%) | 77 (32%) | NS | 14 (33%) | 67 (33%) | NS |
| Dysuria | 2 (20%) | 17 (7%) | NS | 5 (12%) | 14 (7%) | NS |
| Pain | 6 (60%) | 137 (57%) | NS | 25 (59%) | 118 (57%) | NS |

[a]NS = not significant.

TABLE 3. Comparison of physical findings of pregnant women with cervicitis, with and without gonoccocal or chlamydial infection[a]

| | *N. gonorrhoeae* | | | *C. trachomatis* | | |
|---|---|---|---|---|---|---|
| Physical finding | Positive (N = 10) | Negative (N = 238) | *P* | Positive (N = 42) | Negative (N = 206) | *P* |
| Friability | 4 (40%) | 123 (52%) | NS | 23 (55%) | 104 (50%) | NS |
| Mucopus | 5 (50%) | 104 (44%) | NS | 23 (55%) | 86 (42%) | NS |
| Leukorrhea | 8 (80%) | 203 (85%) | NS | 37 (88%) | 174 (84%) | NS |
| *Trichomonas* | 1 (10%) | 20 (8%) | NS | 3 (7%) | 18 (9%) | NS |

[a]NS = not significant.

the most common sexually transmitted bacterial microorganism,[5] with a prevalence from 2 to 37% in pregnant women.[6] *N. gonorrhoeae* was found in approximately 4% of pregnant patients in a study at Parkland Memorial Hospital in Dallas, TX; approximately 46% of gravid women with gonococcal infections were coinfected with *C. trachomatis*.[7]

Braddick et al.[2] examined pregnant women in an antenatal clinic in Nairobi, Kenya, and found that the prevalence of *C. trachomatis* and *N. gonorrhoeae* was 8% and 10%, respectively. The presence of clinical cervicitis (defined as the presence of either endocervical mucopus or induced cervical bleeding) and having more than one sexual partner during the pregnancy were independently predictive of a true cervical infection, with sensitivities and specificities of 61% and 79% and 68% and 93%, respectively. The combination of more than one sexual partner during the pregnancy and the clinical diagnosis of cervicitis raised the specificity to 86%; however, the sensitivity dropped to 11%.

Vuylsteke and colleagues[3] studied 1,160 pregnant women presenting to an antenatal health clinic for initial prenatal care in Kinasha, Zaire. Those patients who were included in the study had symptoms of vaginal discharge or abdominal pain. Nearly

2% of the women tested positive for *N. gonorrhoeae* and 5.2% tested positive for *C. trachomatis.* The factors significantly associated with cervical infections in pregnant women included younger age (mean 22.3 years), single marital status, having had more than one sexual partner in the preceding 12 months, a vaginal discharge, >10 WBCs/hpf in a cervical or vaginal specimen, and a positive leukocyte-esterase dipstick when testing a midstream urine sample. However, no single symptom or physical sign reached an acceptable level of sensitivity and specificity to be predictive of cervical infections. A scoring system based on marital status, maternal age, symptoms of vaginal discharge or abdominal pain, and leukocyte-esterase dipstick of urine was formulated and used in a diagnostic model for the prediction of cervical infections. In their population, they were able to predict infections with a sensitivity of 72% and specificity of 74%; however, the positive predictive value reached only 16%.

Of the variables shown to be associated with cervical *C. trachomatis* infections in nongravid women, only the maternal age was significantly different in our study. We were unable to correlate other clinical signs and symptoms shown to be associated with a positive gonococcal or chlamydial test in nongravid women. Our study revealed no significant association between "clinical cervicitis" in pregnancy and a true cervical infection.

There was a correlation between the diagnosis of lower urinary-tract infection and a subsequent positive test for *C. trachomatis.* Gravid women with this diagnosis were twice as likely to have a cervical chlamydial infection than those without. This finding probably reflects the presence of chlamydia urethritis in these patients[8] and indicates that clinicians should be suspicious of a chlamydial cervical infection in the setting of a lower urinary-tract infection as diagnosed by urinalysis in a pregnant woman.

The ability to clinically diagnose cervical infections due to *N. gonorrhoeae* or *C. trachomatis* may enable clinicians to reduce the maternal and neonatal sequelae of these infections. Our patient population, being primarily indigent, relied heavily upon the emergency room for health care. Therefore, we have chosen to treat all presumptive cases of gonococcal or chlamydial cervical infections (cervicitis), assuming that we may not be able to relay a positive test result and offer treatment once the patient has been discharged from the emergency department. The present study shows that the clinical diagnosis of mucopurulent cervicitis is a poor predictor of endocervical infection with *N. gonorrhoeae* or *C. trachomatis,* with 10/248 (4%) cases positive for *N. gonorrhoeae,* and 42/248 (17%) positive for *C. trachomatis,* for a total of 49/248 (20%) positive for one or the organism. It appears that we overtreat a large number of patients (80%), exposing them to unnecessary antibiotic therapy. The Centers for Disease Control (CDC) recommends the treatment of mucopurulent cervicitis based on the results of testing for *C. trachomatis* and *N. gonorrhoeae,* but cautions that empirical treatment should be given if the likelihood of infection with either of these organisms is high or the patient is not likely to return for treatment.[9]

From the data evaluated, we should have a higher index of suspicion for an endocervical chlamydial infection in pregnancy if the patient is younger than 24 years or has a urinalysis consistent with a lower urinary-tract infection in the setting of clinical cervicitis. There were no identifiable reliable predictors for cervical gonococcal infections. Finally, we should attempt to improve our clinical prediction of gonococcal or chlamydial cervical infections in pregnant women presenting to our emergency department.

As this study evaluated an urban, indigent group of patients, who are at higher risk of cervical infections, this information is only applicable to similar patient populations. By the inclusion criteria for the study, we preselected a group of patients with an inherently higher risk for gonococcal and chlamydial cervical infections. As there were relatively small numbers of patients with positive test results, a subgroup analysis of the data introduces the possibility of a type II error. A larger sample size would help to alleviate this problem and improve the power of the study. A prospective examination of pregnant women with and without the diagnosis of endocervicitis utilizing cultures for *C. trachomatis* and *N. gonorrhoeae* may identify predictors useful for the clinical identification of a true gonococcal or chlamydial endocervical infection in pregnancy.

In summary, the clinical indicators of cervical infection with *C. trachomatis* and *N. gonorrhoeae* were unreliable in our patient population. In light of these findings, continued screening of this patient population for these STDs is recommended.

### REFERENCES

1. Handsfield HH, Jasman LL, Roberts PL, Hanson VW, Kothenbeutel RL, Stamm WE: Criteria for selective screening for *Chlamydia trachomatis* infection in women attending family planning clinics. JAMA 255(13):1730–1734, 1986.
2. Braddick MR, Ndinya-Achola JO, Mirza NB, et al.: Towards developing a diagnostic algorithm for *Chlamydia trachomatis* and *Neisseria gonorrhoeae* cervicitis in pregnancy. Genitourin Med 66:62–65, 1990.
3. Vuylsteke B, Laga M, Alary M, et al.: Clinical algorithms for the screening of women for gonococcal and chlamydial infection: Evaluation of pregnant women and prostitutes in Zaire. Clin Infect Dis 17:82–88, 1993.
4. Cunningham FG, MacDonald PC, Leveno KJ, Gant NF, Gilstrap LC, eds. Sexually transmitted diseases. In: William's Obstetrics. 19th ed. Norwalk, Connecticut: Appleton and Lange, pp 1299–1319, 1993.
5. *Chlamydia trachomatis* infections: Policy guidelines for prevention and control. MMWR 34 (Suppl 3S):53S–73S, 1985.
6. Hardy PH, Hardy JB, Nell EE, et al.: Prevalence of six sexually transmitted disease agents among inner-city adolescents and pregnancy outcome. Lancet 2:333–337, 1984.
7. Christmas JT, Wendel GD, Bawdon RE, Farris R, Cartwright G, Little BB: Concomitant infection with *Neisseria gonorrhoeae* and *Chlamydia trachomatis* infection in pregnancy. Am J Obstet Gynecol 74:295–298, 1989.
8. Stamm WE, Wagner KF, Amsel R, et al.: Causes of the acute urethral syndrome in women. N Engl J Med 303(8):409–415, 1980.
9. Centers for Disease Control and Prevention: 1993 sexually transmitted disease treatment guidelines. MMWR 42(RR-14):49–52, 1993.
